# Myasthenia gravis and pregnancy: clinical implications and neonatal outcome

**DOI:** 10.1186/1471-2474-5-42

**Published:** 2004-11-16

**Authors:** José F Téllez-Zenteno, Lizbeth Hernández-Ronquillo, Vicente Salinas, Bruno Estanol, Orlando da Silva

**Affiliations:** 1 Department of Neurology, National Institute of Medical Sciences and Nutrition. "Salvador Zubirán", Mexico City, Mexico; 2 Department of Pediatrics, University of Western Ontario, London, Ontario, Canada; 3 Neonatology Unit. National Institute of Perinatology, Mexico City, Mexico

## Abstract

**Background:**

The myasthenia gravis is twice as common in women as in men and frequently affects young women in the second and third decades of life, overlapping with the childbearing years. Generally, during pregnancy in one third of patients the disease exacerbates, whereas in two thirds it remains clinically unchanged. Complete remission can occur in some patients.

**Methods:**

To describe the clinical course, delivery and neonatal outcome of 18 pregnant women with the diagnosis of myasthenia gravis. Retrospective chart review of pregnant patients with myasthenia gravis, followed at the National Institute of Perinatology in Mexico City over an 8-year period. Data was abstracted from the medical records on the clinical course during pregnancy, delivery and neonatal outcome.

**Results:**

From January 1, 1996 to December 31, 2003 18 patients with myasthenia gravis were identified and included in the study. The mean ± SD maternal age was 27.4 ± 4.0 years. During pregnancy 2 women (11%) had an improvement in the clinical symptoms of myasthenia gravis, 7 women (39%) had clinical worsening of the condition of 9 other patients (50%) remained clinically unchanged. Nine patients delivered vaginally, 8 delivered by cesarean section and 1 pregnancy ended in fetal loss. Seventeen infants were born at mean ± SD gestational age of 37.5 ± 3.0 weeks and a mean birth weight of 2710 ± 73 g. Only one infant presented with transient neonatal myasthenia gravis. No congenital anomalies were identified in any of the newborns.

**Conclusions:**

The clinical course of myasthenia gravis during pregnancy is variable, with a significant proportion of patients experiencing worsening of the clinical symptoms. However, neonatal transient myasthenia was uncommon in our patient population.

## Background

Myasthenia gravis (MG) is an acquired, neuromuscular, autoimmune disease that presents clinically with weakness and fatigue of the skeletal muscles. The disorder is characterized by a decrease of the number of acetylcholine receptors in the neuromuscular plates, due to an autoimmune process mediated by antibodies directed against the alpha-subunit of the nicotine receptor of the acetylcholine [1]. The disease is twice as common in women as in men and frequently affects young women in the second and third decades of life, overlapping with the childbearing years [2,3]. Generally, during pregnancy in one third of patients the disease exacerbates, whereas in two thirds it remains clinically unchanged [4-8]. Of the women who experience worsening, it usually occurs during the first trimester. Signs and symptoms of MG in pregnant women tend to improve during the second and third trimesters coinciding with the physiological immunosuppression which normally takes place in that period. Complete remission can occur in some patients [4-8].

Papazian [9] reported a 21% incidence of transient neonatal myasthenia gravis (TNMG) on infants born to mothers with MG. In this report 67% of infants developed TNMG within the first few hours after birth and within the first 24 hours of life in 78% of neonates [9]. Onset of TNMG beyond 3 days after birth has not been reported. Two clinical forms of TNMG have been described: typical (71%) and atypical (29%). Clinical features of the atypical form include the presence of arthrogriposis multiplex congenita (AMC) in the fetus or newborn [10]. The severity of AMC in the infant is variable and does not co-relate with neither the severity of maternal MG during pregnancy, or if it is the first or subsequent pregnancies [10].

In anti acetylcholine-receptor (anti-AchR) antibody-associated AMC, fetal or neonatal death is common. The possible mechanisms could be crossing of maternal antibodies through the placenta with consequent blockage of the function of the fetal isoform of the AchR leading to fetal paralysis causing AMC. In the typical form of TNMG the usual clinical findings include poor sucking and generalized hypotonia [11].

Other reported clinical manifestations are week cry (60% to 70%), facial diplegia or paresis (37 to 60%), swallowing and sucking difficulties (50 to 71%), and mild respiratory distress [9-11]. Ptosis (15%) and ophthalmoparesis (8%) are less common. Respiratory distress requiring assisted mechanical ventilation can occur in severe cases (29%) [9,10,12]. Response to an oral or parenteral anticholinesterase agent is usually very good. Complete recovery is expected in less than 2 months in 90% of patients and by 4 months of age in the remaining 10% [9,10,12]. It is not clear why only some babies develop TNMG, but the ability of the mother's serum antibodies to bind to the fetal isoform of the AchR in newborn may be a contributing factor [12].

The purpose of our present study was to report on the clinical course, delivery and neonatal outcome of pregnant women with the diagnosis of myasthenia gravis, followed in our perinatal center.

## Methods

### Patient population

From January 1,1996 to December 31, 2003, 18 pregnant women with MG were treated during pregnancy and delivered at the National Institute of Perinatology, a tertiary referral center in Mexico City, Mexico. The clinical course of the disease during pregnancy, labor and post-partum period was reviewed, as well as the neonatal period in the 17 infants born to MG mothers. All clinical data was ascertained after reviewing and collecting data from the patient's medical records. The diagnosis of myasthenia gravis was made on clinical grounds and confirmed by positive edrophonium chloride and electromyography tests [13,14]. Transient neonatal myasthenia gravis was diagnosed on the bases of clinical signs of generalized hypotonia, sucking disturbances, weak cry and respiratory difficulties.

### Criteria for defining clinical improvement or deterioration

After reviewing the medical records of patients the following criteria was used to define clinical change of MG during pregnancy: a) the first was the type and dosage of medications that the patient received before, during and after pregnancy. Data was collected on the type and doses of medications administered to the patient during the 3 periods, b) the second parameter was the stage of the disease according the Osserman's classification before, during and after pregnancy.

The criteria for improvement, unchanged or worsening of MG during pregnancy were the following: 1) Remission: those patients that presented a total disappearance of the symptoms (Osserman's stage 0) and who did not require any specific medication, 2) Improvement: patients who had clinical improvement of the symptoms and decrease of the dosage of the medications that they received before pregnancy by 30% or more, 3) No change: patients with no clinical change in their symptoms (According to Osserman's classification) and same doses of medications compared with before pregnancy. 4) Deterioration: patients who had a deterioration of the disease (worsening of the Osserman's stage) and an increase in the dosages of medications compared with before the pregnancy, or the need for immunosuppressant drugs such as azathioprine and/or prednisone.

The Osserman's classification used in this study was the one used by the Myasthenia Gravis Foundation of America: grade I: any ocular muscle weakness; grade II: mild weakness affecting other than ocular muscles; III: moderate weakness affecting other than ocular muscles; IV Severe weakness affecting other than ocular muscles; and grade V: Defined by tracheal intubation, with or without mechanical ventilation, except when employed in routine postoperative management [15].

#### Patient follow-up

In the first two trimesters all patients were seen in the clinic once a month, every 15 days between 32 and 36 weeks, and weekly after 36 weeks of gestation. During every visit the dosage of the medications, and the Osserman's stage were reviewed. All patients were seen by a team of obstetricians and clinical neurologists.

### Statistical analysis

Descriptive statistics was used to compute the results.

## Results

During the study period 18 pregnant women with MG were seen at the hospital and had the medical records available for review. The mean ± SD maternal age was 27.4 ± 4.0 years. Before pregnancy 3 patients (17%) were in remission (Osserman's stage 0) and 15 patients (83%) were classified as Osserman's stage II. All the patients were clinically stable before pregnancy. Of the 15 patients with in stage II, 13 (86%) used pyridostigmine, one used pyridostigmine plus steroids (7%), and one used pyridostigmine, azathioprine and steroids. Thymectomy was performed in 17 patients (94%) before the pregnancy. The mean length of time from the start of symptoms to thymectomy was 24.0 months (range: 1–168 months). Other clinical conditions were also diagnosed in 5 patients (28%) before pregnancy: 3 (17%) had impaired glucose tolerance and 2 autoimmune thyroiditis (11%). Serum antibodies against the human acetylcholine receptor assayed by standard RIA were positive in 14 patients (77%). The patients became pregnant at a mean of 2 years post-thymectomy. In our center we prefer to do the thymectomy first and then when the patient is stable we recommend the pregnancy. This is not a generalized protocol in many centers but the majority of our patients were in good conditions before the pregnancy.

During pregnancy 9 patients (50%) did not change the clinical status compared with before pregnancy, 2 (11%) had improvement and 7 (39%) had worsening of the MG. Of the seven patients who deteriorated, one did so in the first trimester and six in the second trimester. Only one patient experienced a myasthenic crisis during pregnancy. During pregnancy 11 patients (61%) received pyridostigmine, one patient (6%) received pyridostigmine plus steroids and another (6%) received pyridostigmine, steroids, and azathioprine. The pregnancy in two patients (11%) was complicated by eclampsy; one woman was diagnosed with chorioamnionitis and another with thrombocytopenia. Pregnancy duration was 37.5 ± 3.0 weeks (range 29–41 weeks). The clinical characteristics of patients is shown in Table 1.

Table 2 shows the delivery mode and neonatal outcome of our patients. Eight patients were delivered by caesarian section. The other ten were delivered by vaginal delivery (one forceps assisted), one of these products was a stillbirth.

Seventeen infants were born at mean ± SD gestational age of 37.5 ± 3.0 weeks and a mean birth weight of 2710 ± 73 g. Only one infant presented with transient neonatal myasthenia gravis. This baby presented with sucking difficulties, which resolved spontaneously by day 7 and did not require any specific treatment. The patient with myasthenic crisis delivered by spontaneous vaginal delivery at 37 weeks. The weight of the newborn was 2800 g without complications. No congenital anomalies were identified in any of the newborns.

## Discussion

Myasthenia gravis is not rare among women of reproductive age, the reported incidence ranges from 1:10,000 to 1:50,000 [3]. Literature describing the clinical course of pregnant myasthenic women mostly consists of single case reports and case series [6-8]. Generally it has been assumed that pregnancy is associated with physiological immunosuppression. There is evidence, as yet unexplained, that polymorphonuclear leukocyte chemotaxis and adherence functions are depressed beginning in the second trimester and continuing during the rest of the pregnancy. It is possible that these depressed leukocyte functions of pregnant women account in part for the improvement observed in some autoimmune diseases. It may also explain the increased susceptibility to certain infections [17]. On the other hand it is well known that some diseases could exacerbate during the pregnancy. This has been reported for example in patients with systemic lupus erythematosus and myasthenia gravis [16,17]. The clinical course of myasthenia gravis in pregnancy is considered to be unpredictable. It has been reported that: a) approximately one third of patients remain the same, one third improve, and the remaining one third worsens, b) the course in one pregnancy does not predict the course in subsequent pregnancies, c) exacerbations occur equally in all three trimesters and 4) therapeutic termination does not demonstrate a consistent benefit in cases of first trimester exacerbation [4,6-9,18].

Schlezinger [4] described the course of MG during pregnancy in 22 myasthenic women with a total of 33 pregnancies. He showed that in one third of the pregnant woman an exacerbation occurred, whereas two thirds showed no change or a remission occurred. In his series the exacerbation usually occurred during the first trimester, with minor clinical changes during the second and third trimesters [4]. Djelmis et al [8] reviewed their experience with 69 pregnancies among 65 women with MG managed over a 28 year-period. 24.6% of patients showed an improvement during the pregnancy, 44.9% did not change and 30.4% suffered exacerbations. In Djelmis et al [8] report the deterioration was observed in the last 4 weeks of pregnancy and in the puerperium. In another study Mitchell et al [6] reported the clinical course of 11 cases of pregnant myasthenia gravis patients. 27.2 % had improvement and 72.7% deteriorated during pregnancy. The deterioration was observed in the third trimester in all patients. One of their patients suffered respiratory failure. They concluded that there were no predictive factors to identify the mother at risk of exacerbation during pregnancy and the risk of neonatal myasthenia gravis. Batochi et al [7] evaluated the course of 47 women who became pregnant after the onset of MG. During pregnancy 42% had no change, 39% improved and 19% got worse. In the experience of Batochi et al [7] the clinical worsening was more frequent in the second trimester and two patients developed respiratory failure. He concluded that the course of the myasthenia gravis during gestation is highly variable and unpredictable and can change in subsequent pregnancies. Recently Picone at al [18] in a series of 12 patients showed worsening in 42% of patients during pregnancy.

Our study showed a frequency of worsening of 33%, being an intermediate frequency compared with the reported frequencies of 15 to 55%. The majority of our patients showed that the worsening occurred in the second trimester as in the Batochi et al [7] series. In the series of Osserman [4] and Djlemis et al [8] the worsening was observed in the third trimester. It is clear from these reports that the clinical course of the disease during pregnancy is highly variable, and difficult to predict. In our study 8 patients had a cesarean section for delivery (47%) and 9 (53%) delivered vaginally (one by forceps extraction). In one patient the pregnancy ended in a stillbirth. Djelmis et al [8] reported vaginal deliver in 82%, Batochi et al [7] in 70%, Mitchel et al [6] in 90% and Picone at al [18] in 58%. Our study showed a rate of cesarean section of 47%, similar to the rate of 42% reported by Picone et al [18]. In a recent study Hoff et al [19] reported the results of a retrospective cohort in Norway. The study population consisted of 127 births to mothers with MG compared with a reference group of 1.9 million births to mothers without MG. They showed that women with MG had a higher rate of complications at delivery, and in particular the risk of preterm rupture of amniotic membranes was three times higher in the MG group compared with the reference group. The rate of interventions during birth was raised and cesarean sections doubled. They concluded that MG is associated with an increased risk of complications during delivery, leading to a higher need for surgical interventions.

Regarding the newborns, our study showed that their weight is lower compared with other studies [6,7]. This may be explained by racial differences between our population and the population reported by others. The incidence of TNMG has been reported between 9 and 30% [6,7] Typical clinical findings in the typical form of TNMG are poor sucking and generalized hypotonia. Other manifestations are swallowing and sucking difficulties and mild respiratory distress. Response to oral or parenteral anticholinesterase agents is usually very good. Complete recovery is expected in less than 2 months in 90% of patients and in the remaining 10% by 4 months [9,10], [12,13]. Only 5% of our patients presented with TNMG, which is less than the rate reported by others. The reason for this lower rate is unexplained but it could be due to genetic variation as suggested by others [9].

In conclusion the present literature in pregnant patients with myasthenia gravis is somewhat limited. It consists mostly of case reports and case series. Our study adds to the body of literature showing that about third of our patients deteriorated during pregnancy, which was observed in the second trimester. Our cesarean section rate was high and the rate of TNMG was relatively low.

## Competing interests

The author(s) declare that they have no competing interests.

## Authors' contributions

JFTZ. Has made substantial contributions to conception and design, or acquisition of data, or analysis and interpretation of data.

LHR. Has made substantial contributions to conception and design, or acquisition of data, or analysis and interpretation of data.

VS. Has made substantial contributions to conception and design, or acquisition of data, or analysis and interpretation of data.

BE. Has been involved in drafting the article or revising it critically for important intellectual content

ODS. Has been involved in drafting the article or revising it critically for important intellectual content

All authors read and approved the final manuscript.

## Pre-publication history

The pre-publication history for this paper can be accessed here:



## Figures and Tables

**Table 1:** Patient characteristics

**Variable**	**Mean ± SD, median (range), # of patients (%)**
**Maternal variables**	
Maternal Age (years)	27.4 ± 4.04
Length of time from diagnosis to thymectomy (months)	24 (1–168)
Period of time from thymectomy to pregnancy (months)	24 (6–36)
Duration of pregnancy (weeks)	37.5 ± 3.0 (29 to 41)
Previous thymectomy (%)	17 (94%)
**Clinical course of disease during pregnancy**	
No change	9 (50%)
Improvement	2 (11%)
Deterioration	7 (39%)

**Table 2:** Delivery and neonatal outcome

**Variable**	**Mean ± SD, # of patients (%)**
**Type of delivery (n = 18)**	
Vaginal (without Forceps)*	9 (50%)
Vaginal (Forceps)	1 (6%)
Caesarian	8 (44%)
**Neonatal outcome (n = 17)**	
Birth weight (g)	2710 ± 73
Neonatal transient myasthenia gravis	1 (6%)

*One vaginal delivery was a stillbirth.
